# Crystal structure of bis­[(5-oxo­oxolan-3-yl)triphen­ylphosphanium] hexa­iodido­tellurate(IV)

**DOI:** 10.1107/S1600536814023940

**Published:** 2014-11-05

**Authors:** Sari M. Närhi, Raija Oilunkaniemi, Risto S. Laitinen

**Affiliations:** a Department of Chemistry, P.O. Box 3000, FI-90014 University of Oulu, Finland

**Keywords:** crystal structure, bis­[triphenyl(5-oxooxolan-3-yl)phosphanium] cation, hexa­iodido­tellurate(2-) anion

## Abstract

The asymmetric unit of the title salt, [C_22_H_20_O_2_P]_2_^+^[TeI_6_]^2−^, consists of one triphenyl(5-oxooxolan-3-yl)phosphanium cation and one half of a hexa­iodido­tellurate(IV) dianion. The Te atom is located at an inversion centre and is octa­hedrally coordinated by six I atoms. The Te—I bond lengths range from 2.9255 (9) to 2.9439 (10) Å. The I—Te—I angles between *cis*-iodide ligands are in the range 87.85 (3)–92.15 (3)°. In the crystal, the components are connected by C—H⋯I inter­actions. In the final refinement of the compound a void of 32 Å^3^ was observed.

## Related literature   

For the isolation and structure of the related compound {PPh_3_(C_4_H_5_O_2_)}_2_[TeI_4_], see: Närhi *et al.* (2013 ▶). For other related structures, see: Srivastava *et al.* (2004 ▶); Närhi *et al.* (2004 ▶). For discussion about the formation of the cation, see: Närhi *et al.* (2013 ▶). 
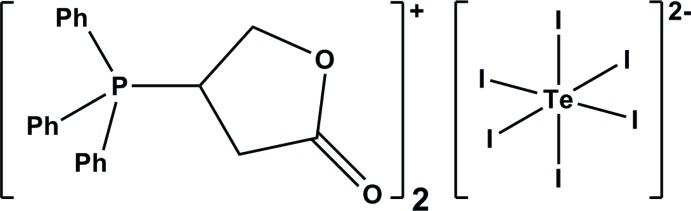


## Experimental   

### Crystal data  

2C_22_H_20_O_2_P^+^·TeI_6_^2−^
*M* *_r_* = 1583.70
Triclinic, 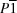

*a* = 9.4479 (19) Å
*b* = 11.022 (2) Å
*c* = 13.259 (3) Å
α = 74.64 (3)°
β = 69.70 (3)°
γ = 77.28 (3)°
*V* = 1236.1 (5) Å^3^
*Z* = 1
Mo *K*α radiation
μ = 4.45 mm^−1^
*T* = 100 K
0.25 × 0.20 × 0.20 mm

### Data collection  

Bruker Nonius KappaCCD diffractometer
Absorption correction: ψ scan (*XPREP* in *SHELXTL*; Sheldrick, 2008 ▶) *T*_min_ = 0.543, *T*_max_ = 0.927
11104 measured reflections
4557 independent reflections
3957 reflections with *I* > 2σ(*I*)
*R*_int_ = 0.039

### Refinement  

*R*[*F* ^2^ > 2σ(*F* ^2^)] = 0.032
*wR*(*F* ^2^) = 0.078
*S* = 1.05
4557 reflections
260 parameters
H-atom parameters constrained
Δρ_max_ = 0.80 e Å^−3^
Δρ_min_ = −1.04 e Å^−3^

### 

Data collection: *COLLECT* (Hooft, 1998 ▶); cell refinement: *DENZO-SMN* (Otwinowski & Minor, 1997 ▶); data reduction: *DENZO-SMN*; program(s) used to solve structure: *SHELXS97* (Sheldrick, 2008 ▶); program(s) used to refine structure: *SHELXL2013* (Sheldrick, 2008 ▶); molecular graphics: *DIAMOND* (Brandenburg, 2006 ▶); software used to prepare material for publication: *WinGX* (Farrugia, 2012 ▶).

## Supplementary Material

Crystal structure: contains datablock(s) I, global. DOI: 10.1107/S1600536814023940/zl2606sup1.cif

Structure factors: contains datablock(s) I. DOI: 10.1107/S1600536814023940/zl2606Isup2.hkl

**Fig. 1.**
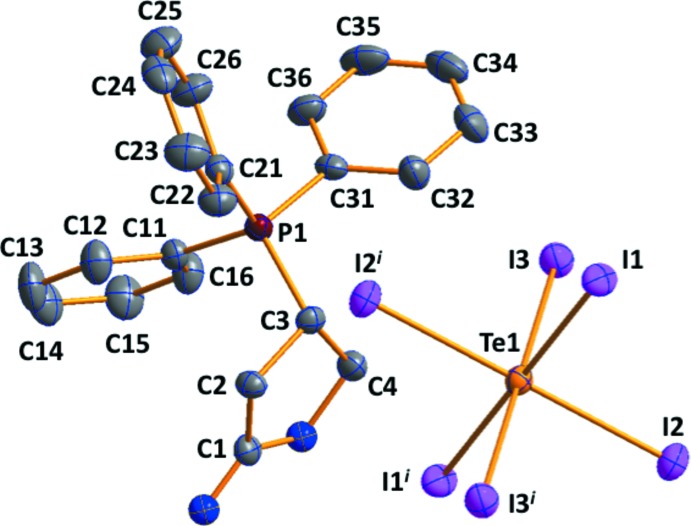
The molecular structure of {Ph3(C4H5O2)P}2[TeI6] indicating the numbering of the atoms. The displacement ellipsoids have been drawn at 50% probability. Hydrogen atoms have been omitted for clarity. Symmetry code: i: -x, -y, -z.

3 4 5 2 2 6 i x y z . DOI: 10.1107/S1600536814023940/zl2606fig1.tif
The mol­ecular structure of {Ph_3_(C_4_H_5_O_2_)P}_2_[TeI_6_] indicating the numbering of the atoms. The displacement ellipsoids have been drawn at 50% probability. Hydrogen atoms have been omitted for clarity. Symmetry code: *i*: −*x*, −*y*, −*z*.

**Fig. 2.**
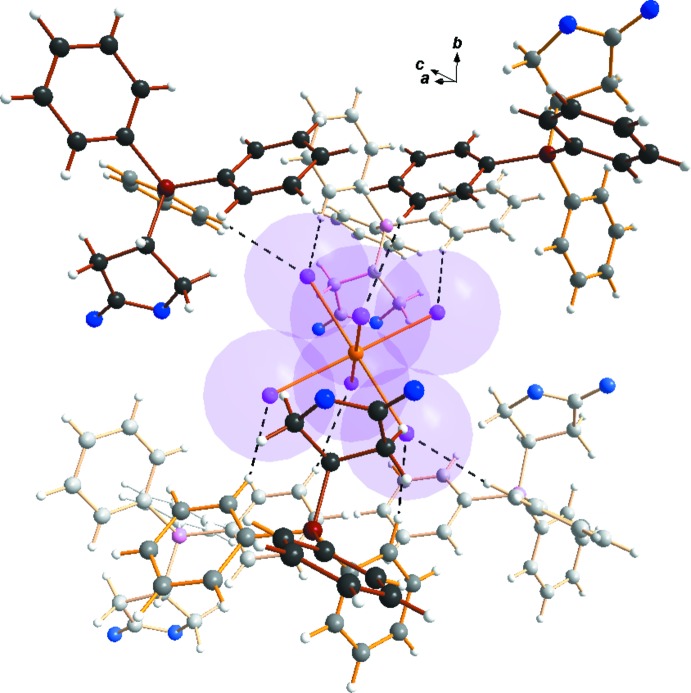
The shortest H···I hydrogen bonds between the cation and the anion. The van der Waals' radius of iodine has been overlaid with the structure of the anion.

. DOI: 10.1107/S1600536814023940/zl2606fig2.tif
The shortest H⋯I hydrogen bonds between the cation and the anion. The van der Waals’ radius of iodine has been overlaid with the structure of the anion.

CCDC reference: 1031805

Additional supporting information:  crystallographic information; 3D view; checkCIF report

## Figures and Tables

**Table 1:** Hydrogen-bond geometry (, )

*D*H*A*	*D*H	H*A*	*D* *A*	*D*H*A*
C35H35I1^ii^	0.95	3.17	4.080(6)	161
C16H16I2	0.95	2.97	3.839(5)	152
C22H22I2^iii^	0.95	3.09	3.875(6)	141
C32H32I3^iii^	0.95	3.08	3.958(6)	155

Symmetry codes: (ii) 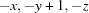; (iii) 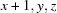.

## supplementary crystallographic information

### S1. Synthesis and crystallization

Dark purple crystals of {PPh_3_(C_4_H_5_O_2_)}_2_[TeI_6_] were isolated in the reaction of Fu_2_Te_2_ (Fu = 2-furyl, C_4_H_5_O), I_2_, and Ph_3_P in THF. Under ambient conditions, the reaction is reported to give a mixture of products (Närhi *et al.* (2013)). The crystals of {PPh_3_(C_4_H_5_O_2_)}_2_[TeI_6_] are probably formed by the reaction of {PPh_3_(C_4_H_5_O_2_)}_2_[TeI_4_] with I_2_. They are formed as a separate layer on the wall of the reaction vessel during slow evaporation of the solvent.

### S2. Refinement

H atoms were positioned geometrically and refined using a riding model with C—H = 0.99 Å and with *U*_iso_(H) = 1.2*U*_eq_(C), 1.00 Å and *U*_iso_(H) = 1.2*U*_eq_(C) and 0.95 Å and *U*_iso_(H) = 1.2*U*_eq_(C) for the methyl­ene, tertiary, and aromatic hydrogens, respectively.

In the final refinement of the compound a void of 32 Å^3^ was observed. The void contains no residual electron density and the volume is very small for solvent molecules. The cavity probably results from the inflexible packing of the bulky, rigid ions of the title compound.

### Crystal data

**Table:**

2C_22_H_20_O_2_P^+^·TeI_6_^2^^−^	*Z* = 1
*M**_r_* = 1583.70	*F*(000) = 736
Triclinic, *P*1	*D*_x_ = 2.128 Mg m^−^^3^
*a* = 9.4479 (19) Å	Mo *K*α radiation, λ = 0.71073 Å
*b* = 11.022 (2) Å	Cell parameters from 3957 reflections
*c* = 13.259 (3) Å	θ = 2.8–25.7°
α = 74.64 (3)°	µ = 4.45 mm^−^^1^
β = 69.70 (3)°	*T* = 100 K
γ = 77.28 (3)°	Block, dark purple
*V* = 1236.1 (5) Å^3^	0.25 × 0.20 × 0.20 mm

### Data collection

**Table:**

Bruker Nonius KappaCCD diffractometer	3957 reflections with *I* > 2σ(*I*)
Radiation source: fine-focus sealed tube	*R*_int_ = 0.039
φ scans, and ω scans with κ offsets	θ_max_ = 25.7°, θ_min_ = 2.8°
Absorption correction: ψ scan (*XPREP* in *SHELXTL*; Sheldrick, 2008)	*h* = −11→11
*T*_min_ = 0.543, *T*_max_ = 0.927	*k* = −13→13
11104 measured reflections	*l* = −15→16
4557 independent reflections	

### Refinement

**Table:**

Refinement on *F*^2^	Secondary atom site location: difference Fourier map
Least-squares matrix: full	Hydrogen site location: inferred from neighbouring sites
*R*[*F*^2^ > 2σ(*F*^2^)] = 0.032	H-atom parameters constrained
*wR*(*F*^2^) = 0.078	*w* = 1/[σ^2^(*F*_o_^2^) + (0.0302*P*)^2^ + 2.1829*P*] where *P* = (*F*_o_^2^ + 2*F*_c_^2^)/3
*S* = 1.05	(Δ/σ)_max_ = 0.001
4557 reflections	Δρ_max_ = 0.80 e Å^−^^3^
260 parameters	Δρ_min_ = −1.04 e Å^−^^3^
0 restraints	Extinction correction: *SHELXL2013* (Sheldrick, 2008), Fc^*^=kFc[1+0.001xFc^2^λ^3^/sin(2θ)]^-1/4^
Primary atom site location: structure-invariant direct methods	Extinction coefficient: 0.0026 (3)

### Special details

**Table:**

Geometry. All e.s.d.'s (except the e.s.d. in the dihedral angle between two l.s. planes) are estimated using the full covariance matrix. The cell e.s.d.'s are taken into account individually in the estimation of e.s.d.'s in distances, angles and torsion angles; correlations between e.s.d.'s in cell parameters are only used when they are defined by crystal symmetry. An approximate (isotropic) treatment of cell e.s.d.'s is used for estimating e.s.d.'s involving l.s. planes.

### Fractional atomic coordinates and isotropic or equivalent isotropic displacement parameters (Å^2^)

**Table:**

	*x*	*y*	*z*	*U*_iso_*/*U*_eq_	
C1	0.4386 (5)	−0.0474 (4)	0.3688 (4)	0.0261 (10)	
C2	0.5254 (5)	0.0617 (4)	0.3411 (4)	0.0249 (10)	
H2A	0.6366	0.0328	0.3197	0.030*	
H2B	0.4965	0.1035	0.4044	0.030*	
C3	0.4790 (5)	0.1532 (4)	0.2435 (4)	0.0228 (9)	
H3	0.5725	0.1785	0.1830	0.027*	
C4	0.4013 (6)	0.0707 (4)	0.2060 (4)	0.0273 (10)	
H4A	0.3070	0.1196	0.1909	0.033*	
H4B	0.4708	0.0425	0.1384	0.033*	
C11	0.1952 (5)	0.2509 (4)	0.4010 (4)	0.0244 (10)	
C12	0.2007 (6)	0.2481 (6)	0.5045 (4)	0.0384 (12)	
H12	0.2854	0.2737	0.5123	0.046*	
C13	0.0825 (7)	0.2079 (6)	0.5970 (4)	0.0461 (15)	
H13	0.0872	0.2058	0.6678	0.055*	
C14	−0.0408 (6)	0.1711 (6)	0.5871 (5)	0.0434 (14)	
H14	−0.1222	0.1456	0.6508	0.052*	
C15	−0.0464 (6)	0.1714 (6)	0.4833 (5)	0.0437 (14)	
H15	−0.1304	0.1442	0.4760	0.052*	
C16	0.0711 (6)	0.2116 (5)	0.3913 (4)	0.0351 (12)	
H16	0.0672	0.2123	0.3205	0.042*	
C21	0.4479 (5)	0.3983 (4)	0.3112 (4)	0.0253 (10)	
C22	0.5921 (6)	0.3606 (5)	0.3220 (4)	0.0343 (12)	
H22	0.6433	0.2775	0.3143	0.041*	
C23	0.6625 (7)	0.4450 (5)	0.3441 (5)	0.0438 (14)	
H23	0.7619	0.4195	0.3512	0.053*	
C24	0.5882 (7)	0.5647 (5)	0.3555 (4)	0.0394 (13)	
H24	0.6362	0.6215	0.3713	0.047*	
C25	0.4456 (7)	0.6029 (5)	0.3442 (5)	0.0444 (14)	
H25	0.3952	0.6861	0.3522	0.053*	
C26	0.3745 (6)	0.5213 (5)	0.3213 (5)	0.0386 (13)	
H26	0.2761	0.5485	0.3124	0.046*	
C31	0.2836 (5)	0.3777 (4)	0.1675 (4)	0.0256 (10)	
C32	0.3746 (6)	0.3662 (5)	0.0613 (4)	0.0349 (12)	
H32	0.4692	0.3115	0.0505	0.042*	
C33	0.3278 (7)	0.4342 (5)	−0.0284 (5)	0.0420 (14)	
H33	0.3890	0.4241	−0.1004	0.050*	
C34	0.1938 (7)	0.5157 (5)	−0.0138 (5)	0.0439 (14)	
H34	0.1633	0.5632	−0.0757	0.053*	
C35	0.1024 (7)	0.5292 (5)	0.0909 (6)	0.0467 (15)	
H35	0.0095	0.5860	0.1004	0.056*	
C36	0.1458 (6)	0.4600 (5)	0.1820 (5)	0.0365 (12)	
H36	0.0823	0.4686	0.2539	0.044*	
O1	0.3662 (4)	−0.0376 (3)	0.2954 (3)	0.0303 (7)	
O2	0.4316 (4)	−0.1369 (3)	0.4452 (3)	0.0392 (9)	
P1	0.35088 (13)	0.29555 (11)	0.28121 (10)	0.0218 (3)	
Te1	0.0000	0.0000	0.0000	0.02328 (12)	
I1	0.22939 (4)	0.16601 (3)	−0.14044 (3)	0.03310 (11)	
I2	−0.06205 (4)	0.14116 (3)	0.17266 (3)	0.03253 (11)	
I3	−0.23996 (4)	0.16762 (3)	−0.08546 (3)	0.03373 (12)	

### Atomic displacement parameters (Å^2^)

**Table:**

	*U*^11^	*U*^22^	*U*^33^	*U*^12^	*U*^13^	*U*^23^
C1	0.024 (2)	0.027 (2)	0.023 (2)	−0.0060 (19)	−0.0004 (19)	−0.005 (2)
C2	0.027 (3)	0.022 (2)	0.027 (2)	−0.0056 (19)	−0.011 (2)	−0.0030 (19)
C3	0.018 (2)	0.022 (2)	0.026 (2)	−0.0016 (18)	−0.0037 (18)	−0.0059 (19)
C4	0.029 (3)	0.026 (2)	0.026 (2)	−0.003 (2)	−0.010 (2)	−0.004 (2)
C11	0.024 (2)	0.027 (2)	0.023 (2)	−0.0039 (19)	−0.0071 (18)	−0.0047 (19)
C12	0.033 (3)	0.055 (3)	0.031 (3)	−0.013 (2)	−0.008 (2)	−0.010 (3)
C13	0.045 (3)	0.071 (4)	0.023 (3)	−0.019 (3)	−0.003 (2)	−0.010 (3)
C14	0.035 (3)	0.056 (4)	0.033 (3)	−0.016 (3)	0.001 (2)	−0.005 (3)
C15	0.032 (3)	0.056 (4)	0.043 (3)	−0.021 (3)	−0.003 (2)	−0.009 (3)
C16	0.030 (3)	0.049 (3)	0.030 (3)	−0.014 (2)	−0.007 (2)	−0.012 (2)
C21	0.026 (2)	0.026 (2)	0.022 (2)	−0.0076 (19)	−0.0056 (19)	−0.0022 (19)
C22	0.030 (3)	0.031 (3)	0.042 (3)	−0.007 (2)	−0.011 (2)	−0.006 (2)
C23	0.037 (3)	0.041 (3)	0.058 (4)	−0.013 (2)	−0.019 (3)	−0.007 (3)
C24	0.052 (4)	0.040 (3)	0.032 (3)	−0.027 (3)	−0.012 (2)	−0.001 (2)
C25	0.053 (4)	0.033 (3)	0.052 (4)	−0.008 (3)	−0.016 (3)	−0.015 (3)
C26	0.036 (3)	0.030 (3)	0.051 (3)	0.000 (2)	−0.014 (3)	−0.015 (2)
C31	0.029 (3)	0.019 (2)	0.030 (3)	−0.0039 (18)	−0.013 (2)	−0.0010 (19)
C32	0.036 (3)	0.036 (3)	0.027 (3)	−0.001 (2)	−0.008 (2)	−0.004 (2)
C33	0.051 (4)	0.045 (3)	0.029 (3)	−0.016 (3)	−0.013 (2)	0.006 (2)
C34	0.061 (4)	0.033 (3)	0.045 (3)	−0.012 (3)	−0.034 (3)	0.007 (3)
C35	0.051 (4)	0.028 (3)	0.069 (4)	0.008 (2)	−0.038 (3)	−0.009 (3)
C36	0.037 (3)	0.029 (3)	0.045 (3)	0.005 (2)	−0.018 (2)	−0.011 (2)
O1	0.0299 (18)	0.0295 (17)	0.0345 (19)	−0.0091 (14)	−0.0131 (15)	−0.0034 (15)
O2	0.051 (2)	0.0310 (19)	0.034 (2)	−0.0133 (17)	−0.0117 (17)	0.0003 (17)
P1	0.0209 (6)	0.0212 (6)	0.0223 (6)	−0.0030 (4)	−0.0060 (5)	−0.0036 (5)
Te1	0.0205 (2)	0.0252 (2)	0.0228 (2)	−0.00338 (16)	−0.00399 (16)	−0.00623 (17)
I1	0.02691 (19)	0.03040 (18)	0.0364 (2)	−0.00844 (13)	−0.00070 (14)	−0.00623 (14)
I2	0.03118 (19)	0.0377 (2)	0.03201 (19)	−0.00362 (14)	−0.00814 (14)	−0.01573 (15)
I3	0.02802 (19)	0.0372 (2)	0.03218 (19)	0.00223 (14)	−0.00940 (14)	−0.00627 (15)

### Geometric parameters (Å, º)

**Table:**

C1—O2	1.208 (6)	C22—C23	1.394 (8)
C1—O1	1.341 (6)	C22—H22	0.9500
C1—C2	1.498 (6)	C23—C24	1.371 (8)
C2—C3	1.547 (6)	C23—H23	0.9500
C2—H2A	0.9900	C24—C25	1.368 (8)
C2—H2B	0.9900	C24—H24	0.9500
C3—C4	1.549 (7)	C25—C26	1.381 (8)
C3—P1	1.826 (4)	C25—H25	0.9500
C3—H3	1.0000	C26—H26	0.9500
C4—O1	1.446 (6)	C31—C32	1.394 (7)
C4—H4A	0.9900	C31—C36	1.397 (7)
C4—H4B	0.9900	C31—P1	1.785 (5)
C11—C12	1.383 (7)	C32—C33	1.386 (7)
C11—C16	1.392 (7)	C32—H32	0.9500
C11—P1	1.792 (5)	C33—C34	1.366 (9)
C12—C13	1.388 (8)	C33—H33	0.9500
C12—H12	0.9500	C34—C35	1.384 (9)
C13—C14	1.371 (8)	C34—H34	0.9500
C13—H13	0.9500	C35—C36	1.388 (8)
C14—C15	1.395 (8)	C35—H35	0.9500
C14—H14	0.9500	C36—H36	0.9500
C15—C16	1.381 (7)	Te1—I2^i^	2.9255 (9)
C15—H15	0.9500	Te1—I2	2.9255 (9)
C16—H16	0.9500	Te1—I1	2.9417 (12)
C21—C22	1.380 (7)	Te1—I1^i^	2.9417 (12)
C21—C26	1.398 (7)	Te1—I3	2.9439 (10)
C21—P1	1.797 (5)	Te1—I3^i^	2.9439 (10)
			
O2—C1—O1	121.0 (4)	C25—C24—H24	119.7
O2—C1—C2	127.2 (5)	C23—C24—H24	119.7
O1—C1—C2	111.8 (4)	C24—C25—C26	120.3 (5)
C1—C2—C3	104.3 (4)	C24—C25—H25	119.8
C1—C2—H2A	110.9	C26—C25—H25	119.8
C3—C2—H2A	110.9	C25—C26—C21	119.7 (5)
C1—C2—H2B	110.9	C25—C26—H26	120.1
C3—C2—H2B	110.9	C21—C26—H26	120.1
H2A—C2—H2B	108.9	C32—C31—C36	119.2 (5)
C2—C3—C4	103.4 (3)	C32—C31—P1	119.4 (4)
C2—C3—P1	113.1 (3)	C36—C31—P1	121.2 (4)
C4—C3—P1	111.7 (3)	C33—C32—C31	120.3 (5)
C2—C3—H3	109.5	C33—C32—H32	119.8
C4—C3—H3	109.5	C31—C32—H32	119.8
P1—C3—H3	109.5	C34—C33—C32	120.2 (6)
O1—C4—C3	106.2 (4)	C34—C33—H33	119.9
O1—C4—H4A	110.5	C32—C33—H33	119.9
C3—C4—H4A	110.5	C33—C34—C35	120.3 (5)
O1—C4—H4B	110.5	C33—C34—H34	119.9
C3—C4—H4B	110.5	C35—C34—H34	119.9
H4A—C4—H4B	108.7	C34—C35—C36	120.3 (5)
C12—C11—C16	119.2 (5)	C34—C35—H35	119.8
C12—C11—P1	120.5 (4)	C36—C35—H35	119.8
C16—C11—P1	120.2 (4)	C35—C36—C31	119.6 (5)
C11—C12—C13	120.0 (5)	C35—C36—H36	120.2
C11—C12—H12	120.0	C31—C36—H36	120.2
C13—C12—H12	120.0	C1—O1—C4	111.5 (4)
C14—C13—C12	120.7 (5)	C31—P1—C11	110.7 (2)
C14—C13—H13	119.6	C31—P1—C21	109.2 (2)
C12—C13—H13	119.6	C11—P1—C21	108.3 (2)
C13—C14—C15	119.8 (5)	C31—P1—C3	107.7 (2)
C13—C14—H14	120.1	C11—P1—C3	109.6 (2)
C15—C14—H14	120.1	C21—P1—C3	111.2 (2)
C16—C15—C14	119.4 (5)	I2^i^—Te1—I2	180.0
C16—C15—H15	120.3	I2^i^—Te1—I1	91.74 (3)
C14—C15—H15	120.3	I2—Te1—I1	88.26 (3)
C15—C16—C11	120.8 (5)	I2^i^—Te1—I1^i^	88.26 (3)
C15—C16—H16	119.6	I2—Te1—I1^i^	91.74 (3)
C11—C16—H16	119.6	I1—Te1—I1^i^	180.0
C22—C21—C26	119.6 (5)	I2^i^—Te1—I3	87.85 (3)
C22—C21—P1	122.3 (4)	I2—Te1—I3	92.15 (3)
C26—C21—P1	118.1 (4)	I1—Te1—I3	92.00 (3)
C21—C22—C23	119.7 (5)	I1^i^—Te1—I3	88.00 (3)
C21—C22—H22	120.1	I2^i^—Te1—I3^i^	92.15 (3)
C23—C22—H22	120.1	I2—Te1—I3^i^	87.85 (3)
C24—C23—C22	120.1 (5)	I1—Te1—I3^i^	88.00 (3)
C24—C23—H23	120.0	I1^i^—Te1—I3^i^	92.00 (3)
C22—C23—H23	120.0	I3—Te1—I3^i^	180.0
C25—C24—C23	120.5 (5)		
			
O2—C1—C2—C3	−174.2 (5)	P1—C31—C36—C35	175.4 (4)
O1—C1—C2—C3	6.8 (5)	O2—C1—O1—C4	−174.9 (4)
C1—C2—C3—C4	−14.0 (5)	C2—C1—O1—C4	4.2 (5)
C1—C2—C3—P1	107.0 (4)	C3—C4—O1—C1	−13.4 (5)
C2—C3—C4—O1	16.6 (4)	C32—C31—P1—C11	−148.4 (4)
P1—C3—C4—O1	−105.3 (3)	C36—C31—P1—C11	35.8 (5)
C16—C11—C12—C13	−0.8 (8)	C32—C31—P1—C21	92.4 (4)
P1—C11—C12—C13	−177.1 (5)	C36—C31—P1—C21	−83.4 (4)
C11—C12—C13—C14	−0.3 (9)	C32—C31—P1—C3	−28.5 (5)
C12—C13—C14—C15	1.4 (10)	C36—C31—P1—C3	155.7 (4)
C13—C14—C15—C16	−1.4 (9)	C12—C11—P1—C31	−146.6 (4)
C14—C15—C16—C11	0.4 (9)	C16—C11—P1—C31	37.2 (5)
C12—C11—C16—C15	0.7 (8)	C12—C11—P1—C21	−26.8 (5)
P1—C11—C16—C15	177.0 (4)	C16—C11—P1—C21	157.0 (4)
C26—C21—C22—C23	0.8 (8)	C12—C11—P1—C3	94.7 (4)
P1—C21—C22—C23	179.3 (4)	C16—C11—P1—C3	−81.5 (4)
C21—C22—C23—C24	0.2 (8)	C22—C21—P1—C31	−129.0 (4)
C22—C23—C24—C25	−0.7 (9)	C26—C21—P1—C31	49.5 (5)
C23—C24—C25—C26	0.1 (9)	C22—C21—P1—C11	110.3 (4)
C24—C25—C26—C21	1.0 (9)	C26—C21—P1—C11	−71.3 (4)
C22—C21—C26—C25	−1.4 (8)	C22—C21—P1—C3	−10.3 (5)
P1—C21—C26—C25	−179.9 (4)	C26—C21—P1—C3	168.2 (4)
C36—C31—C32—C33	−1.0 (8)	C2—C3—P1—C31	−170.7 (3)
P1—C31—C32—C33	−176.8 (4)	C4—C3—P1—C31	−54.5 (4)
C31—C32—C33—C34	1.7 (9)	C2—C3—P1—C11	−50.1 (4)
C32—C33—C34—C35	−1.2 (9)	C4—C3—P1—C11	66.0 (4)
C33—C34—C35—C36	−0.1 (9)	C2—C3—P1—C21	69.6 (4)
C34—C35—C36—C31	0.8 (8)	C4—C3—P1—C21	−174.2 (3)
C32—C31—C36—C35	−0.3 (8)		

Symmetry code: (i) −*x*, −*y*, −*z*.

### Hydrogen-bond geometry (Å, º)

**Table:**

*D*—H···*A*	*D*—H	H···*A*	*D*···*A*	*D*—H···*A*
C35—H35···I1^ii^	0.95	3.17	4.080 (6)	161
C16—H16···I2	0.95	2.97	3.839 (5)	152
C22—H22···I2^iii^	0.95	3.09	3.875 (6)	141
C32—H32···I3^iii^	0.95	3.08	3.958 (6)	155

Symmetry codes: (ii) −*x*, −*y*+1, −*z*; (iii) *x*+1, *y*, *z*.

## References

[bb1] Brandenburg, K. (2006). *DIAMOND*. Crystal Impact GmbH, Bonn, Germany.

[bb2] Farrugia, L. J. (2012). *J. Appl. Cryst.* **45**, 849–854.

[bb3] Hooft, R. W. W. (1998). *COLLECT*. Nonius BV, Delft, The Netherlands.

[bb4] Närhi, S. M., Malo, K., Oilunkaniemi, R. & Laitinen, R. S. (2013). *Polyhedron*, **65**, 308–315.

[bb5] Närhi, S. M., Oilunkaniemi, R., Laitinen, R. S. & Ahlgrén, M. (2004). *Inorg. Chem.* **43**, 3742–3750. 10.1021/ic049789v 15180431

[bb6] Otwinowski, Z. & Minor, W. (1997). *Methods in Enzymology*, Vol. 276, *Macromolecular Crystallography*, Part A, edited by C. W. Carter Jr & R. M. Sweet, pp. 307–326. New York: Academic Press.

[bb7] Sheldrick, G. M. (2008). *Acta Cryst.* A**64**, 112–122. 10.1107/S0108767307043930 18156677

[bb8] Srivastava, P. C., Bajpai, S., Bajpai, S., Ram, C., Kumar, R., Jasinski, J. P. & Butcher, R. J. (2004). *J. Organomet. Chem.* **689**, 194–202.

