# Supplementary figures and images for: Crystal structure of bis­[(5-oxo­oxolan-3-yl)triphen­ylphosphanium] hexa­iodido­tellurate(IV)
Source: Acta Crystallogr Sect E Struct Rep Online. 2014 Nov 5;70(Pt 12):o1241. doi: 10.1107/S1600536814023940 (PMC4257451; doi:10.1107/S1600536814023940)

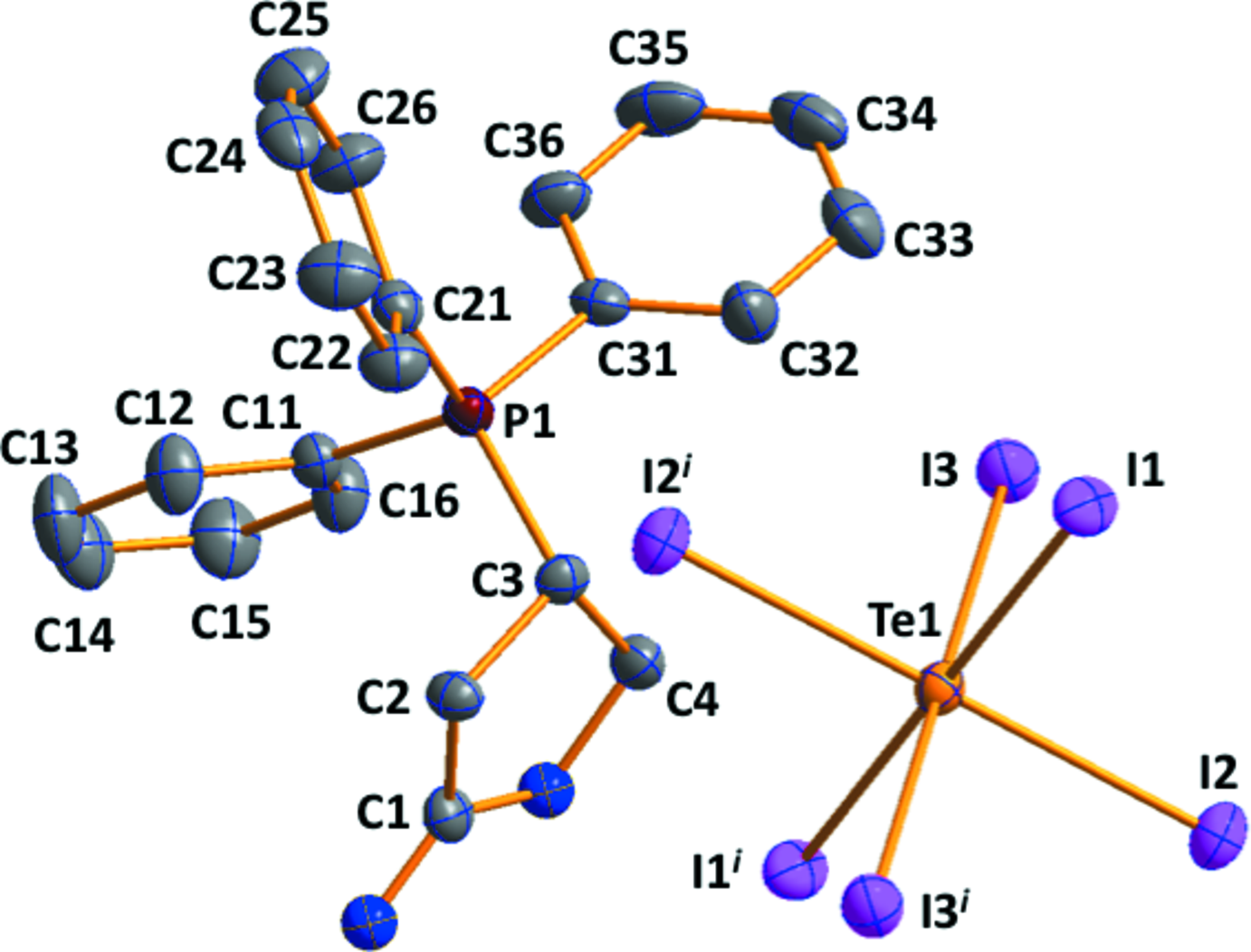

Supplement: Supplementary file 3 [file e-70-o1241-fig1.tif]

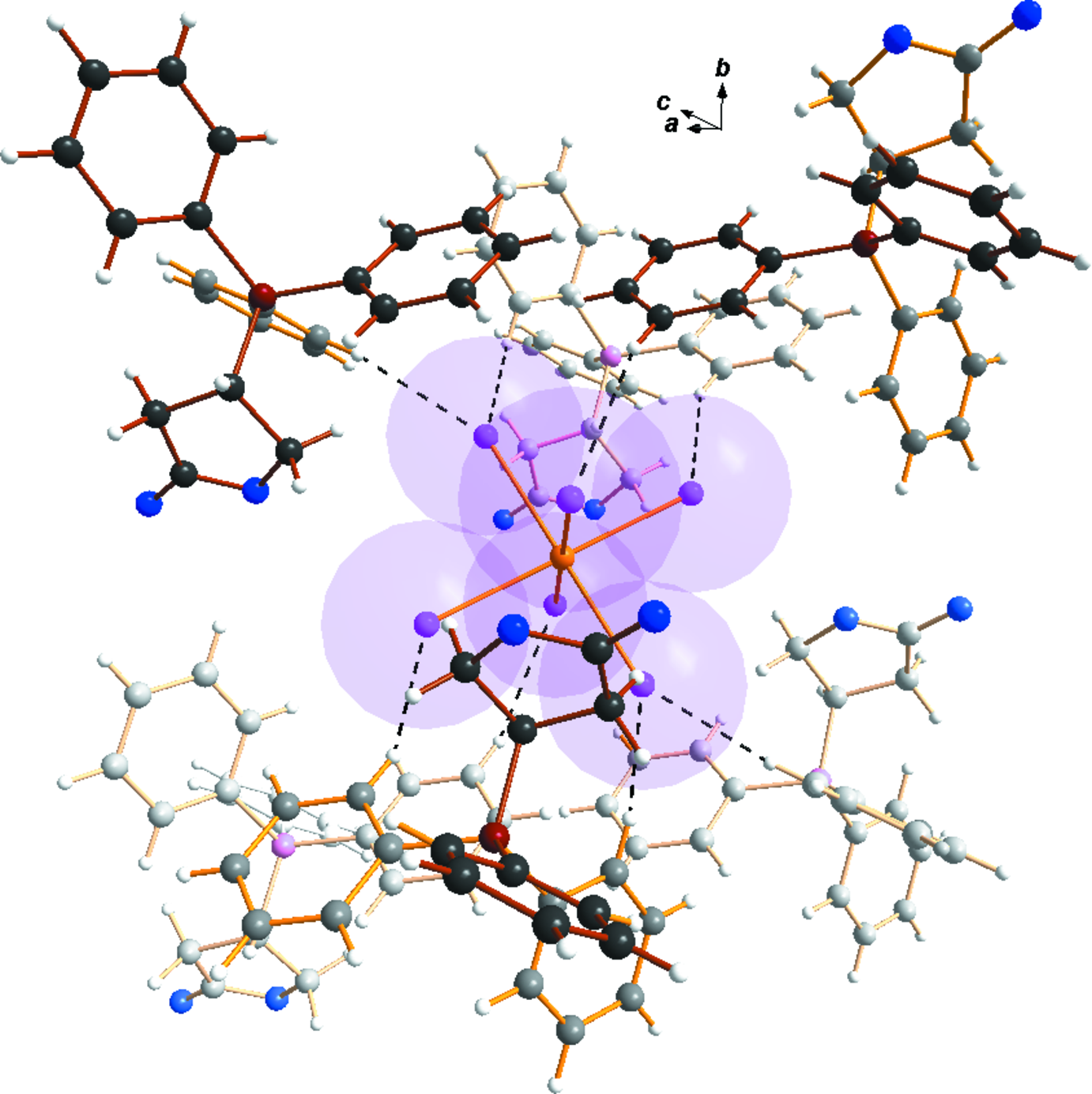

Supplement: Supplementary file 4 [file e-70-o1241-fig2.tif]
